# Novel Targets for Drug Use in Eosinophilic Granulomatosis With Polyangiitis

**DOI:** 10.3389/fmed.2021.754434

**Published:** 2021-11-02

**Authors:** Martina Uzzo, Francesca Regola, Barbara Trezzi, Paola Toniati, Franco Franceschini, Renato Alberto Sinico

**Affiliations:** ^1^Department of Clinical and Experimental Sciences, University of Milano-Bicocca, Monza, Italy; ^2^Nephrology and Dialysis Unit, Ospedale San Gerardo di Monza, Monza, Italy; ^3^Department of Clinical and Experimental Sciences, University of Brescia, Brescia, Italy; ^4^Rheumatology and Clinical Immunology Unit, ASST-Spedali Civili of Brescia, Brescia, Italy

**Keywords:** Eosinophilic Granulomatosis with Polyangiitis, heart involvement, novel therapies, biologics, rituximab, mepolizumab, omalizumab

## Abstract

Eosinophilic Granulomatosis with Polyangiitis (EGPA) is a rare autoimmune disease characterized by medium and small vessels inflammation. Cardiac vasculitic involvement is one of the most severe manifestations with a significant impact on patients' long-term prognosis: anyway, a specific therapeutic approach for heart involvement in EGPA has not been explored yet. Current regimen consists of a long-term therapy with high dose of glucocorticoids, causing the well-known related-adverse events; immunosuppressive drugs are used in patients with severe manifestations, with some limitations. New therapeutic approaches are needed for patients with refractory disease or contraindications to conventional therapies. The quest for the ideal therapy is going toward a more and more personalized approach: on the one hand, efforts are made to use already existing therapies in the most appropriate way; on the other hand, new insights into EGPA pathogenesis allow the discovery of new targets, as demonstrated by mepolizumab and rituximab, targeting eosinophils, and B-cell compartments. This review summarizes the emerging therapies used in EGPA, focusing on the most recent studies on biologics and analyzing their efficacy and safety.

## Introduction

As classified by the 2012-revised Chapel Hill consensus conference, Eosinophilic Granulomatosis with Polyangiitis (EGPA, formerly Churg Strauss syndrome) is a rare systemic necrotizing vasculitis of small and medium size vessels, characterized by asthma and blood and tissue eosinophilia; among all kind of vasculitis, EGPA can have an impressive heart involvement (1). It is a rare disease with a prevalence ranging between 7.3 and 17.8 per million and an annual incidence of 0.9–2.4 per million (2–4).

EGPA belongs to the anti-neutrophil cytoplasm antibody (ANCA)-associated vasculitis group (AAV), including granulomatosis with polyangiitis (GPA), and microscopic polyangiitis (MPA). However, only 30–40% of patients are ANCA positive and ~70% with myeloperoxidase (MPO) specificity: the ANCA positive disease is characterized by vasculitic features and shares pathogenetic mechanisms with the other AAV; the ANCA negative disease seems to share the pathogenetic mechanisms observed in eosinophilic syndromes instead (5). A recent genome-wide association study confirmed the existence of those two phenotypes from a genetical point of view: the former is linked with HLA-DQ, the latter with genes involved in mucosal responses (6). The natural history of the disease involves three steps: asthma or allergy related symptoms, followed by eosinophilia and lung infiltrates and finally, after a mean of 9.3 years, vasculitic manifestations (7).

## CV Insights

Heart involvement is one of the most severe manifestations in EGPA and its primary cause of death (31%), due to myocardial infarction, cardiac insufficiency, or arrhythmia. Myocardial involvement is associated with a higher mortality risk rate according to the Five Factor Score (FFS), a prognostic tool: as described in the FFS published in 1996, cardiac insufficiency showed a marked risk of mortality in EGPA [HR 2.8; 95% CI 0.15–0.9; *P* = 0.03; (8)]. The pathophysiological mechanisms underlining CV involvement are coronary vasculitis, extravascular granulomas and eosinophilic interstitial infiltrate, causing eosinophilic myocarditis, pericarditis, hypertension, valvulopathy, and congestive heart failure (9, 10).

Cardiac involvement in EGPA is often related to a specific clinical phenotype, characterized by ANCA negativity and eosinophilic infiltrates. The ANCA positive phenotype is characterized by vasculitic features, with peripheral neuropathy, purpura, renal involvement, and biopsy-proven vasculitis; less frequently, heart involvement can be present [5.7% in the ANCA positive phenotype vs. 22.4% in the ANCA negative one, *P* = 0.042; (11)]. While the ANCA -positive phenotype is associated with more frequent relapses (35.2 vs. 22.5%, *P* = 0.01), the ANCA-negative one is characterized by a worst prognosis with a higher mortality [5.6 vs. 12.5%, *P* < 0.5; (9)].

Despite clinical and prognostic differences, current EGPA treatment is not based specifically on heart involvement nor on the ANCA-phenotype, while emerging strategies aim at a more personalized approach. Screening strategies aiming at the early recognition and treatment of EGPA patients with cardiac involvement are still not defined (12). From 62 to 90% of patients in disease remission have cardiac changes; furthermore, from 50 to 65% of EGPA patients have late gadolinium enhancement (83% sensitivity, 56% specificity) on cardiac MRI during active disease. In this context, echocardiography could be a cheap and safe method to investigate CV involvement (83% sensitivity and 80% specificity in EGPA): according to the recently published American College of Rheumatology (ACR) guidelines, an echocardiogram at the time of diagnosis is recommended for all patients, even in the absence of cardiac symptoms (13). New studies are warranted to compare different imaging tests and define a common screening program (14–16).

## The Therapeutic Challenge in Eosinophilic Granulomatosis With Polyangiitis

The establishment of a proper treatment plan in EGPA is a challenging decision and a risk benefit balance of the aimed treatment should be considered. The new published guidelines for EGPA reflect the evolving management of the disease: on the one hand, new therapeutic approaches are needed to treat relapsing and/or refractory disease; on the other hand, novel therapies aim at reducing GC exposure over time and therefore its drug-induced toxicity (13).

### Eosinophilic Granulomatosis With Polyangiitis: Current Therapy

EGPA is an extremely rare disease: its current treatment is mainly based on studies involving the others, more frequent AAV.

Topical drugs should be preferentially used to treat asthma and ENT manifestations: these symptoms, which greatly impact on the patient's quality of life, could be easily alleviated by local therapies and their course is usually independent from the vasculitic systemic manifestations (17).

EGPA therapy with systemic involvement, as well as of MPA and GPA, needs to be staged in an induction and a maintenance phase (18).

According to Comarmond et al., although almost 90% of EGPA patients achieved remission, 25.3% relapsed, and 18% experienced asthma or ENT flares, justifying prolonged use of GC (9).

Therapeutic strategies differ according to disease severity, defined as life-threatening manifestations, and/or organ impairment associated with long-term poor prognosis. EGPA severity can be assessed with the Five Factor Score (FFS), a prognostic tool published in 1996 and revised in 2011 in a broader population of patients. The 2011 version included 5 items: age (>65 years), heart involvement, GI involvement (hemorrhage, infarction, or pancreatitis), renal insufficiency with a stabilized peak creatininemia (>150 μmol/L) and lack of ENT manifestations (19).

While the definitions of severe and non-severe EGPA used in the recent published ACR guidelines were not based on the FFS, the tool was found to be useful in clinical practice to facilitate treatment decisions. Anyway, its applicability to newer therapies is still unknown (13).

For active and severe EGPA, defined as FFS ≥1 or other life-and organ threatening manifestations (alveolar hemorrhage, eye involvement, and fulminant mononeuritis multiplex), IV pulses (7.5–15 mg/kg/day) or high dose oral GC should be administered as initial therapy. Prednisone should be taken at 1 mg/kg/day for 2–3 weeks, followed by gradual tapering (ideally down to 0.3 mg/kg/day after 3 months and 0.15 mg/kg/day after 6 months), to the minimal effective dose.

Duration and tapering of the GC therapy have not been defined yet in RCT for EGPA: the optimum daily dose to avoid adverse events should be <7.5 mg (18); however, the 85% of patients need a long-term GC therapy to control severe asthma and ENT manifestations. Potential GC toxicity is a major concern, especially in young patients needing treatment for several years or often for the whole life (9).

The adjunction of a cytotoxic agent is recommended to treat severe EGPA: cyclophosphamide (CYC) has been commonly used as the first line induction regimen. Data on the use of CYC as induction regimen comes from studies regarding others AAV: according to the CYCLOPS trial, bolus-IV CYC appeared to be the safest induction therapy as compared to the oral-daily administration because of the reduced cumulative dose allowing less adverse events (20, 21); the CORTAGE trial included 14 patients with EGPA and proposed a low dose IV CYC regimen (0.5 mg/m^2^ every 2–3 weeks until remission), showing no differences and fewer adverse events as compared to the standard regimen (22). The actual dose is 0.5–0.7 g/m^2^: 3 infusions every 2 weeks and 3 infusions every 3 weeks (a total of 6 infusions). According to the new published ACR guidelines, either CYC or RTX may be prescribed for remission induction in severe EGPA, thanks to the increasing experience with others AAV. Given the higher clinical experience, CYC should be preferentially used in patients with active cardiac involvement and a worst prognosis. Anyway, the comparative effectiveness of CYC and RTX as induction regimen in EGPA is still unknown. Regardless of the induction regimen used, RTX is also recommended as induction regimen in relapsing disease, in order to reduce the dose related CYC toxicity (13).

Without maintenance therapy relapse rates range between 73.8 and 85.7%, depending on the CYC therapy duration (6 or 12 months) (23): maintenance therapy should be started 2 weeks after last CYC administration. Once again, no RCT has compared the available maintenance therapy in EGPA, neither has defined its duration: the azathioprine and methotrexate equivalence and the superiority to mycophenolate mofetil in other AAV has been stated in RCT (24, 25). Azathioprine is administered at a dose of 2 mg/Kg/day and methotrexate at a dose of 0.25 mg/Kg/week. Despite the lack of data, a 18–24 months maintenance therapy after remission is recommended (13).

In patients with non-severe EGPA (FFS = 0) the addiction of an immunosuppressive to the GC therapy was previously not recommended (26–28); anyway, according to the new published ACR guidelines, the addition of an adjunctive immunosuppressive regimen is recommended to lower GC toxicity. Particularly, treatment with mepolizumab is the first line choice, as its efficacy was recently stated in a RCT; on the other hand, the well-known methotrexate, azathioprine, and mycophenolate mofetil have not been assessed in RCTs for this purpose. Switch between immunosuppressive treatments is recommended for relapsing disease. GC alone can be used in appropriate patients (mild asthma, allergic symptoms, pregnancy) (13).

### Further Therapies

The use of plasmapheresis (PLEX) in EGPA is based on data coming from other AAV studies: PLEX is considered for patients with rapidly progressive glomerulonephritis or interalveolar hemorrhage (29). The long-term follow-up did not show a sustained effect of PLEX in terms of risk reduction of the composite outcome of death and ESRD (30). Furthermore, a small RCT involving 14 EGPA patients did not show any benefit in adding PLEX to the ongoing therapy (31).

Data on the intravenous immunoglobulins (IVIg) role in EGPA is scarce: according to case reports, IVIg can be used in a case-by-case evaluation as a second line therapy, especially in patients with myocardial or neural involvement (32, 33).

Interferon a (IFNa) has a cytoreductive action on eosinophils and seems to induce EGPA remission in several case reports. Anyway, it has limited efficacy on major relapses and has important AEs (34, 35).

### Eosinophilic Granulomatosis With Polyangiitis: New Targeted Therapies

Since EGPA shares features with systemic AAV, eosinophilic disorders and asthma, new biologics for non-responding, and relapsing disease are needed for induction and maintenance of vasculitis remission and asthma targeting therapies.

A deep knowledge of EGPA pathogenesis allows the identifications of new targets for drug use: the emerging role of eosinophils and the Th2 interleukins (ILs) activation pathway led the way to the identification of new therapies targeting eosinophils biology, such as the anti-IL-5 mepolizumab; the role of B cell compartment in EGPA has not been completely cleared, but the anti-CD20 monoclonal antibody rituximab has been extensively studied in the others AAV and its use in EGPA is currently under investigation.

#### Anti-B-cells: Rituximab (Anti-CD20)

Rituximab (RTX) is a chimeric anti-CD20 monoclonal antibody: CD20 is a B cell membrane specific antigen involved in B cell differentiation and B-T cell stimulation; it is expressed by all lineage of B cells except for pro-B cells and plasma cells (36). Because of its B cell depletion activity, RTX was firstly approved in 1997 for treatment of B cell lymphoma and in 2005 for rheumatoid arthritis (37, 38).

One case report in 2001 firstly motivated the study of RTX as an induction regimen for GPA and MPA, reporting the efficacy of peripheral CD20 positive B cells depletion and a satisfactory clinical response (39). RTX is now approved as both induction and maintenance regimen in GPA and MPA treatment, thanks to RCT suggesting non-inferiority compared to CYC in general AAV, the reduction of costs due to the availability of biosimilars and the better safety profile compared to CYC. Anyway, no EGPA patients were included in those studies (40, 41).

RTX use in AAV is based on the pathogenicity of ANCA antibodies: the clear pathogenetic role of MPO- ANCA in EGPA was shown *in vitro* and mouse models (42, 43).

The depletion of progenitors of ANCA producing plasma-cells is not the only explanation for RTX efficacy: other B cells functions are involved in AAV pathogenesis (44).

IL-5 production in B-T cell interaction plays a key role in eosinophils maturation and survival, showing a strong association with clinical parameters of EGPA activity [Birmingham Vasculitis Activity Score (BVAS), eosinophilia] (45). Higher levels of CD80+, CD27+, CD95+ B cells, and lower levels of CD20+ B cells were described in patients with frequently relapsing EGPA (46). Despite only 30% of EGPA patients are ANCA-positive, overlap exists between the two phenotypes: particularly, IgG4 immune response is always present. IgG4 serum concentration, as a surrogate of B-lymphocyte activation, reflects disease activity, and IgG4 B cells massively infiltrates the organs involved (47, 48). Furthermore, the activity of CYC on B cells confirms their pathogenetic role (49). Differently from CYC, RTX acts directly toward its target, preserving other functions of the immune system. The recommended dose for AAV remission induction is 375 mg/m^2^/week for 4 weeks; for rheumatoid arthritis the standard dose is 1 g every 2 weeks, approved for AAV treatment too. The maintenance dose is 500 mg every 6 months for almost 18 months (50, 51).

Stated the role of B immune response in EGPA pathogenesis, many case reports, and open label studies suggested the efficacy of RTX in EGPA treatment: in a recent retrospective collaborative study, 63 patients with relapsing/refractory EGPA treated with RTX were collected from different centers around Europe. In 87% of patients RTX was administered for a vasculitis flare mainly characterized by peripheral neuropathy, skin and renal manifestation; the 83% of patients had active asthma at the treatment start. The BVAS declined from a median of 8.5 (IQR 5–13) at start to 1 (IQR 0–4.5) at 6 months and to 0 (IQR 0–2) at 12 months; remission rate, partial response, treatment failure, and adverse events requiring treatment stop were 49, 24, 24, and 3%, respectively. Remission rate was better in ANCA-positive patients compared to ANCA negative, without a statistically significant difference. The sparing GC effect was significant but not complete, from a median of 20 mg/day (IQR 15–37.5) at baseline to 7.5 mg/day (IQR 5–10) both at 6 and 12 months, due to the asthma long term therapies (52).

In another retrospective single-center study, two groups of 14 patients, matched for sex, and age, were treated with RTX or CYC as induction regimens and followed-up for 3 years. The 86% of RTX treated patients had a relapsing/remitting disease. Remission rates were not statistically significant between the two treatment groups (OR = 1.39; 95% CI: 0.28–6.84; *P* = 0.404). RTX was also associated with GC sparing from a median of 22.5–5 mg in 12 months. Furthermore, in the RTX group there was a trend toward more ANCA-positive patients achieving remission compared to ANCA-negative (45 vs. 23%) (53). Those data are concordant with previous studies, showing high rates of improvement and reduced need of GC after RTX therapy, especially in ANCA-positive patients, with a lower effect on asthma and ENT symptoms (54, 55).

Furthermore, in a AAV RCT, a RTX based maintenance therapy was associated with a lower risk of relapse than azathioprine, including renal relapse. In the 5-year extended follow-up, RTX was still superior, although in the context of a high relapse risk after therapy withdrawal (50). Concerning EGPA, a scheduled maintenance RTX therapy significantly reduced the relapse rate, as compared to on demand administration (56).

RTX has a similar safety profile as described in RCT for other AAV (27, 28, 50, 54). Two phase 3 RCT are currently ongoing to evaluate a RTX based induction (REOVAS) and maintenance regimen (MAINRITSEG) compared to conventional therapeutic strategy in patients with newly diagnosed or relapsing EGPA (ClinicalTrials.gov Identifier: NCT02807103; ClinicalTrials.gov Identifier: NCT03164473).

In conclusion, RTX effectively induced and sustained remission in patients with a new diagnosis or a refractory/remitting EGPA; it also worked as a GC sparing agent, with a potential benefit among ANCA-positive patients. Further RTCs are needed to confirm these data.

#### Anti-Th2-ILs

##### Mepolizumab (Anti-IL-5)

Cytokines and chemokines have a key role in the regulation pathway of other cells of the immune system. Particularly, IL-5 is the major cytokine responsible for eosinophils activation and survival: it is produced both by Th2 and innate lymphoid cells, regulating innate immunity (57).

Mepolizumab is an anti-IL-5 humanized monoclonal IgG1 antibody which prevents IL-5 binding with the a-subunit of its receptor, mainly expressed on eosinophils. It was firstly approved for the treatment of severe eosinophilic asthma (58).

Thanks to its involvement in the allergic pathway, mepolizumab was investigated in many allergic-related diseases, such as hypereosinophilic syndromes, atopic dermatitis, and chronic rhinosinusitis. EGPA shares the pathogenetic mechanisms observed in eosinophilic syndromes, too. The eosinophils proliferation in EGPA causes massive tissue toxicity due to eosinophils products: eosinophils cationic protein and eosinophils peroxidase are found at high levels in sera, urine and tissues (59). Furthermore, EGPA eosinophils seems to have a lower expression of pro-apoptotic genes (BLCL13, CASP2, CARD4) and defective CD95 (Apo-1 Fas)-mediated apoptosis (60, 61). As seen in asthma, the Th2 lymphocyte phenotype is activated: EGPA sera and bronchoalveolar lavage (BAL) fluid contains high concentrations of IL-5, IL-4, and IL-13 (62). The elevated concentration of IL-5 in EGPA patients suggests this cytokine as a potential target of therapy.

After some open-label pilot studies showing a potential benefit of mepolizumab in EGPA (63, 64), a double-blinded RTC involving 136 patients with uncontrolled non-severe disease (asthma and/or ENT manifestations or mostly non-severe systemic vasculitis), compared the addiction of mepolizumab or placebo to the target therapy. All patients were taking GC and a half were using other immunosuppressive therapy. Mepolizumab was injected subcutaneously at a dose of 300 mg monthly up to week 48, three times the dose approved for asthma. Remission was defined as a BVAS of 0 and no more than 4 mg of prednisone/day; active asthma was considered a feature of relapse. Only 10% of patients were ANCA positive. In the mepolizumab arm 28% of patients obtained a sustained remission for at least 24 weeks, as opposed to 3% of placebo-arm patients (OR 5.91 (95%CI 2.68–13.03; *p* < 0.001). Relapses were less common in the mepolizumab group at 52 weeks (56 vs. 82%, HR 0.32 (95%CI 0.21–0.5; *p* < 0.01), with fewer flares involving both vasculitic and asthma features. Asthma symptoms regressed, without changing in lung-function-tests. Forty-four percent of subjects treated with mepolizumab were able to taper prednisolone or prednisone to <4 mg/day, compared with 7% of subjects who received placebo during weeks 48 through 52. No differences were found between the two arms according to adverse events, previously transient, and not severe (65).

This trial led to the FDA approval of mepolizumab in 2017 as the first specific drug for EGPA. Mepolizumab is now considered a potential treatment for non-severe relapsing/refractory EGPA, with limited data on life-threatening manifestations; particularly, the specific impact on vasculitic features is still unclear, as symptoms were combined with asthma and ENT manifestations.

Long term efficacy is currently under evaluation in an extension phase of the first trial: patients who previously required a dose of prednisolone (or equivalent) of ≥5 mg/day for adequate control of their EGPA were included (104 patients are enrolled), receiving subcutaneously administered mepolizumab at a dose of 300 mg every 4 weeks (ClinicalTrials.gov Identifier: NCT03298061).

While mepolizumab was approved at the dosage of 100 mg/month subcutaneously for the treatment of severe eosinophilic asthma, the approved FDA dose for EGPA is 300 mg/month. The use of mepolizumab in Europe at this dosage is currently off-label: real-life data are scares and its optimal dosage and route of administration is still unclear. While previous studies reported higher doses of IV mepolizumab (750 mg/month) (66, 67), recent data report positive results with low-dose mepolizumab (100 mg/month): low-dose mepolizumab showed clinically relevant benefit in exacerbation rates, asthma symptoms, GC, and immunosuppressive use in EGPA patients (68).

In a retrospective European collaborative study, remission rates at 12 months in patients receiving MEPO at a dose of 100 and 300 mg were 76 and 82%, respectively, with a comparable GC sparing effect: low-dose MEPO could be used as a first line therapy, with the possibility to increase to 300 mg monthly in cases with inappropriate response, since 10% of patients showed improvement after dose escalation. Anyway, further studies are needed to validate the low-dose and standard-dose benefits for the control of systemic and respiratory symptoms in EGPA (68).

##### Future Therapies: Anti-IL-5, Anti-IL-4, and Anti-IL-13

Further anti-IL-5 therapies are currently under investigation: after the successful completion of phase 3 RCTs in asthma, reslizumab (anti-IL-5), and benralizumab (anti-IL-5 a receptor) were investigated in phase 2 trials. Reslizumab was well-tolerated and resulted in a significant reduction in daily oral GC (*P* < 0.05). Benralizumab was also well-tolerated, allowing an oral GC reduction, and reduced exacerbations in EGPA. Larger controlled trials are warranted to evaluate the role of both therapies in EGPA (69, 70).

All Th2 ILs are involved in EGPA pathogenesis: IL-4 and IL-13 are also involved in Th2 activation. Recent and ongoing trials for asthma may open new possibilities for EGPA treatment (58): dupilumab is a fully human monoclonal antibody that binds to the alpha subunit of the IL-4 receptor, inhibiting the activity of both IL-13 and IL-4. It is currently approved for uncontrolled moderate-to-severe atopic dermatitis, moderate to severe asthma, and inadequately controlled chronic rhinosinusitis with nasal polyposis.

#### Anti-IgE: Omalizumab

IgE are involved in the allergic pathway shared by EGPA. Omalizumab is a monoclonal IgG antibody which recognizes free circulating IgE, preventing the binding to its specific high-affinity receptor and interfering with mast cells and basophils degranulation. The beneficial effect of omalizumab in EGPA concerns the downregulation if the IgE receptor, lowering mast cells activation and their interaction with eosinophils. Furthermore, the IgE-mediated antigen presenting processes and the Th2 amplification of inflammatory reactions is inhibited (71). Omalizumab is currently used for the treatment of severe asthma with elevated IgE, chronic spontaneous urticaria and allergic rhinitis (72–74). The drug is injected subcutaneously every 2–4 weeks, based on body weight and serum IgE, with a good safety profile.

Available information on its use in EGPA is contradictory and scarce. First, the drug was mostly used in patients with severe EGPA associated asthma resistant to GC: while some reports showed positive results, with a GC sparing effect, information about vasculitic involvement is scarce. The dose used was different among studies, without a scheduled regimen.

The available evidence supports the use of omalizumab as maintenance therapy in EGPA patients with uncontrolled and severe asthma/ENT symptoms but with a complete control of non-allergic symptoms. Anyway, in one discordant case report, two patients suffered from asthma exacerbations (75). Furthermore, other studies suggested an association between omalizumab intake in severe asthma and the onset of EGPA: while a role of omalizumab as a trigger factor of EGPA cannot be excluded, the steroid sparing could reveal a hidden disease (76–79).

## Conclusions

The quest for the ideal regimen in EGPA is going toward a more personalized approach, looking for new therapies as well as tailored regimens adapted to different subsets of patients (divided according to disease severity, age, organ involvement, and predictable outcomes) (7). A deep knowledge of the pathogenesis of EGPA allows the identifications of new targets for drugs use (Table 1). The recent guidelines highlight gaps in our knowledge for the treatment of EGPA: new studies are warranted to find more reliable markers and indicators of disease activity, to identify the best use (dose, duration, long-term safety) of actual therapies and to investigate new targeted therapies, with steroid sparing activity.

**Table 1:** Main biological therapies in EGPA (36–68, 71–79).

**Drug**	**Pathogenetic basis**	**Evidence in EGPA**	**Dose**	**Clinical use**
Rituximab (36–56)	*Anti-CD20—B cell differentiation and B-T cell stimulation* ◼ ANCA pathogenetic antibodies ◼ Eosinophils maturation and survival promoted by IL-5 (B-T cell interaction) ◼ IgG4 (a surrogate of B-lymphocyte activation) infiltration of the organs involved	◼ Case reports and open label studies ◼ Previous AAV studies (not involving EGPA) ◼ Two phase 3 RCT ongoing in EGPA: *- REOVAS* (RTX as induction therapy) (NCT02807103) *- MAINRITSEG* (RTX as maintenance therapy) (NCT03164473)	◼ Induction: 375 mg/m^2^/week for 4 weeks or 1 g every 2 weeks (AAV treatment) ◼ Maintenance: 500 mg every 6 months for almost 18 months	*RCTs ongoing:* ◼ New diagnosis or refractory/remitting disease ◼ GC sparing agent
Mepolizumab (57–68)	*Anti-IL-5—eosinophils activation and survival* ◼ Eosinophils products: massive tissue toxicity ◼ EGPA eosinophils: lower expression of pro-apoptotic genes and defective apoptosis	◼ *FDA approval in 2017 as the first specific drug for EGPA* (RTC involving 136 patients with uncontrolled non-severe disease) ◼ Long term efficacy: ongoing RCT (NCT03298061)	◼ 300 mg/month (FDA approved) ◼ 100 mg/month (severe eosinophilic asthma, under evaluation in EGPA)	◼ Non-severe relapsing/refractory disease
Omalizumab (71–79)	*Anti-free circulating IgE* ◼ Lower mast cells activation and interaction with eosinophils ◼ Inhibition of Th2 and IgE mediated antigen presenting processes	*Evidence contradictory and scarce:* ◼ Positive results in EGPA with asthma resistant to GC ◼ Scarce information about vasculitic involvement ◼ Possible trigger factor for EGPA	◼ Subcutaneously every 2–4 weeks	◼ Maintenance therapy in patients with uncontrolled and severe asthma/ENT symptoms but with a complete control of non-allergic symptoms

## Author Contributions

FF and RAS conceived the study. MU and FR conducted a review of the literature and drafted the manuscript. MU, FR, BT, PT, FF, and RAS reviewed and edited the manuscript and support the study. All authors checked the final version of the manuscript.

## Conflict of Interest

RAS has received consulting fees from GSK, Roche, and Otsuka. The remaining authors declare that the research was conducted in the absence of any commercial or financial relationships that could be construed as a potential conflict of interest.

## Publisher's Note

All claims expressed in this article are solely those of the authors and do not necessarily represent those of their affiliated organizations, or those of the publisher, the editors and the reviewers. Any product that may be evaluated in this article, or claim that may be made by its manufacturer, is not guaranteed or endorsed by the publisher.
